# Preparation of high-crystalline and non-metal modified g-C_3_N_4_ for improving ultrasound-accelerated white-LED-light-driven photocatalytic performances

**DOI:** 10.1038/s41598-023-41473-y

**Published:** 2023-09-12

**Authors:** Abdolreza Tarighati Sareshkeh, Mir Saeed Seyed Dorraji, Zhaleh Karami, Saeedeh Shahmoradi, Elnaz Fekri, Hoda Daneshvar, Mohammad Hossein Rasoulifard, Denis N. Karimov

**Affiliations:** 1 Applied Chemistry Research Laboratory, Department of Chemistry, Faculty of Science, University of Zanjan, Zanjan, Iran; 2 Federal Scientific Research Center “Crystallography and Photonics”, Russian Academy of Sciences, Leninsky Prospekt 59, 119333 Moscow, Russia

**Keywords:** Chemical engineering, Environmental sciences, Chemistry, Materials science, Nanoscience and technology

## Abstract

As a non-metallic organic semiconductor, graphitic carbon nitride (g-C_3_N_4_) has received much attention due to its unique physicochemical properties. However, the photocatalytic activity of this semiconductor faces challenges due to factors such as low electronic conductivity and limited active sites provided on its surface. The morphology and structure of g-C_3_N_4_, including macro/micro morphology, crystal structure and electronic structure can affect its catalytic activity. Non-metallic heteroatom doping is considered as an effective method to tune the optical, electronic and other physicochemical properties of g-C_3_N_4_. Here, we synthesized non-metal-doped highly crystalline g-C_3_N_4_ by one-pot calcination method, which enhanced the photocatalytic activity of g-C_3_N_4_ such as mesoporous nature, reduced band gap, wide-range photousability, improved charge carrier recombination, and the electrical conductivity was improved. Hence, the use of low-power white-LED-light illumination (λ ≥ 420 nm) and ultrasound (US) irradiation synergistically engendered the Methylene Blue (MB) mineralization efficiency elevated to 100% within 120 min by following the pseudo-first-order mechanism under the following condition (i.e., pH 11, 0.75 g L^−1^ of O-doped g-C_3_N_4_ and S-doped g-C_3_N_4_, 20 mg L^−1^ MB, 0.25 ml s^−1^ O_2_, and spontaneous raising temperature). In addition, the rapid removal of MB by sonophotocatalysis was 4 times higher than that of primary photocatalysis. And radical scavenging experiments showed that the maximum distribution of active species corresponds to superoxide radical ${\mathrm{O}}_{2}^{\cdot-}$. More importantly, the sonophotocatalytic degradation ability of O-doped g-C_3_N_4_ and S-doped g-C_3_N_4_ was remarkably sustained even after the sixth consecutive run.

## Introduction

Until now, lives dependent on the water environment and human being have faced various crises due to the enormous consumption of dyes in various industries such as textiles, biology, pharmaceutical, cosmetics, food packaging, analytical chemistry, plastic-derived chemicals, and other aspects of daily life. In these manufactories, approximately 15% of dyes are released into water resources without any purification process^1, 2^. Methylene blue (MB) has various applications and is widely used in diverse fields like biological, medical, pharmaceutical and chemical industries. The release of methylene blue in wastewater effluent has harmful environmental effects on water, living organisms and fishes. Therefore, it seems necessary to remove such hazardous compounds from wastewater effluent.

As a promising technology, advanced Oxidation Processes (AOPs) have attracted increasing attention due to their capability to generate the reactive radicals to eliminate water pollution^3^. Among the well-known processes of AOPs, conventional sonolysis is suitable for the degradation of persistent organic pollutants. The sonolysis process is basically based on the sonoluminescence process and production of “hot spots” due the ultrasonic cavitation. The hot spots produced during the ultrasonic cavitation cause the direct generation of Hydroxyl radicals (^·^OH) from water pyrolysis and the indirect formation of ^·^OH through reacting Hydrogen radical (^·^H) with ${\mathrm{O}}_{2}^{\cdot-}$^1, 4^. Using the high-frequency US alone consumes a large amount of money, time, and energy. Studies have shown that the combination between catalytic particles and sonolysis, as sonocatalysis, has attracted attention due to its outstanding advantages, including stable performance, uncomplicated equipment, cost-effectiveness, and the creation of more hot spots on catalyst surfaces^5, 6^.

When sonocatalysis is combined with photocatalysis, this is known as sonophotocatalysis which can increase the efficiency of pollutant degradation. Alongside heat generation from collapsing microbubbles, the sonoluminescence phenomenon cause to emit long- and short-wavelength irradiations that are highly profitable for exciting electron from the valance band (VB) to the conduction band (CB) of semiconductors with narrow- and broad-energy bandgap (Eg), respectively^6–8^. The photocatalysts, also have markedly light-absorption capabilities for extraordinary production of various ROS. Like other AOPs, photocatalysis is unable to degrade resistant compounds with high efficiency, this problem is caused by strong tendency of photocatalysts to aggregate, low light absorption ability, and recombination of charge carriers. Hence, low frequency US (20–40 kHz) can be associated with photocatalysis. As a result, hybrid AOPs could reinforce the additional quantities of e^–^h^+^ pairs, prevent the accumulation of electrons in CB, inhibit the creation of charge recombination centers, etc^9–11^.

Among the available light sources, light emitting diodes (LED) have recently attracted much attention. LEDs are semiconductors that can be used as an alternative light source for light-dependent applications such as photocatalytic detoxification/decontamination of air and water environments. Therefore, low-power LEDs have unique characteristics, which include strong and almost concentrated radiation, low electrical energy consumption, small size, simple intensity adjustment, free of mercury and harmful metals, flexibility for design. Different types of photocatalytic reactors with good quality. Controlled lighting conditions, no need for a cooling system in the reactor, and a longer half-life compared to conventional light sources^12, 13^. So far, many researchers have tried to use LED photocatalysts in drinking water purification systems as well as pollutant degradation. LEDs have poor homogeneity in the light spectrum emitted at full power. Meanwhile, high-power LEDs (more than 200 W) face challenges: (i) Traditional lamps generate too much heat, but heat generation by high power LEDs cannot be ignored. (ii) The emission time decreases with time, so that 50,000 h is the lifetime of the light, which is about 5 times longer than that of the mercury vapor lamp^14^. Therefore, it is very important that a photocatalyst has sufficient ability to absorb the emitted light in concert with the LEDs used until pollutant removal with high efficiency^15^.

Among the carbon nitride family, graphitic carbon nitride g-C_3_N_4_-based materials has attracted great attention due to their unique structural features. The sustainable fabrication of g-C_3_N_4_-based materials has wide applications in the fields of energy conversion and storage, such as photocatalytic evolution of H_2_, photocatalytic evolution of O_2_, photocatalytic water splitting, photoreduction of CO_2_ source, electrocatalytic H_2_ evolution, and O_2_ evolution^16^. The g-C_3_N_4_ is a polymeric-based and metal free conjugated semiconductor known as the “holy grail” for visible-light-driven photocatalysis; and, it can be synthesized by cost-effective, simple, earth-abundant, and environmentally friendly precursors and methods. The unique p-conjugated g-C_3_N_4_ network causes photocatalytic advantages such as narrow Eg (less than 2.9 eV) and physicochemical strength of this compound^17–19^. Despite these advantages, pure g-C_3_N_4_ (CN) has some disadvantages such as the high recombination rate of charge carriers, limited light-harvesting ability, low specific surface area, low electrical conductivity, and bulky massive topology. These disadvantages can be overcome by using different modulation methods, e.g., Elemental doping, crystal nature change, combination of noble metals, construction of heterogeneous structures, and morphology transformation^20–22^.

The low crystallinity and insufficient renewability are attributed to the incomplete polymerization of Nitrogen-rich precursors. To solve this issue, the synthesis of g-C_3_N_4_ crystalline forms can effectively reduce the population of –NH_x (x = 1 or 2)_ and improve the photocatalytic performance in the visible region^21–23^. In addition, the synthesis of CN with low crystallinity creates a high specific surface area, which reduces the absorption capacity and reduces the photocatalytic ability^24^. Also, high charge mobility can be provided by high crystal preparation of g-C_3_N_4_^25, 26^.

Recent studies show that the use of melamine precursor to produce graphite carbon nitride compared to urea and cyanuric acid has advantages such as: (I) reducing the number of stretching vibrations of uncondensed amino N–H bonds, and reducing pollutant absorption. (ii) increased crystallinity. (III) increasing photo absorption in the visible light region. However, the bulk morphology and low active surface in graphitic carbon nitride prepared by melamine compared to other precursors lead to limitations in the catalytic performance of this semiconductor^27^. The next parameter that affects the degree of polycondensation of melamine is calcination temperature. Calcination temperature affects the crystallinity, morphology, light absorbing ability and porosity of graphite carbon nitride. For example, when the calcination temperature is higher than 500 °C, it leads to: (i) production of extensive nitrogen-rich carbon nitride with a layered structure. (ii) Organized crystal morphology with large flakes. (iii) Band gap suitable for use in the visible light region. (iv) increase in BET surface area^28^. Most importantly, when the calcination time increases, it increases the size of the CN network^28, 29^. One of the leading topics that has attracted the attention of researchers is the development of catalysts that have new vacancies in their structure. CN has two types of vacancies including carbon and nitrogen. And introducing non-metallic elements such as components containing sulfur, oxygen or phosphorus into its network can improve the photochemical and electrocatalytic properties and also promote the separation and migration of charge carriers^30–35^. In general, it seems that calcining at high temperature, using impurity materials with melamine and increasing the calcination time can improve the crystallinity, electronic, morphological and chemical properties of graphite carbon nitride particles.

As above mentioned, it is not cost-effective to use high power light or US sources. In this paper, we have developed two different novel photocatalysis by preparing the high-crystalline and non-metal-doped g-C_3_N_4_. The physicochemical features of the catalysts were investigated by various characterization tools e.g., FE-SEM, FT-IR, EDS, PL, HR-TEM, XRD, SAED pattern, BET-BJH, UV–vis DRS, Zeta-potential DLS, and EIS. On this basis, the sonophotocatalytic performances were assessed under the low-power white-LED-light illumination and US irradiation.

## Experimental

### Reagents and materials

Melamine (99.9%), Thiourea (98%), Ethanol (96%), Hydrochloric Acid (HCl (37%)), Sodium Hydroxide (NaOH (> 98.5%)), Oxalic acid (AO), anhydrous Sodium Sulfate (Na_2_SO_4_ (99%)), 1-Butanol (BA), para-Benzoquinone (BQ), and Polyethylene Glycol ((PEG) M_W_ = 20,000 g mol^−1^) were supplied from Merck Company. MB (λ_max_ = 664 nm) was bought from the Dr-Mojallali Company. The materials were used without further purification.

### Synthesis of photocatalysts

Graphitic carbon nitride was prepared according to the method presented in our previous report^36^. In detail proper amount of melamine was heated at 520 °C in a crucible with a cover for 2 h. The as prepared yellow powder then washed by ethanol for three times and denoted as CN.

In order to prepare O doped g-C_3_N_4_, 10 g melamine and 6 g Oxalic acid were dissolved in 50 ml solution mixing of distilled water and ethanol; under continuous stirring magnetically at 80 °C. The heating process continued until the complete removal of the solvent. The obtained sample was calcined in a ceramic crucible with a lid from room temperature to 520 °C at a heating rate of 5 °C/min for 2 h. after that the product cooled to room temperature and were ground to a fine powder. The resulting sample was labeled as (OCN)^37^.

To produce g-C_3_N_4_, 10 g of thiourea and 10 g of melamine were calcined in a crucible with a lid at 520°C with a heating rate of 5°C for 2 h and then cooled to room temperature. The prepared sample then washed with water and ethanol and denoted as (SCN)^38^.

### Characterization and instrumentation

The X-ray Diffraction (XRD; Bruker D8 with Cu 40 Kα and 30 mA, USA) was used to study the crystallinity of the samples. Field Emission Scanning Electron Microscopy (FE-SEM; TESCAN, MIRA3, France) was applied to discern the surface topology, the Energy-Dispersive X-ray Spectroscopy (EDS) was executed to detect the elemental components in the samples. Brunauer–Emmett–Teller (BET) and Barrett–Joyner–Halenda (BJH) were utilized to measure the specific surface area (S_BET_), pore size, and pore volume via the N_2_-gas adsorption–desorption method (BEL, Belsorp-mini-II analyzer, Japan), which degassing and operating temperature were + 200 and −196 °C, respectively. Dynamic Light Scattering ((DLS), Malvern, DLS zeta-sizer ZS90 model, United Kingdom) was executed to determine the Zeta (ζ) potential and the average particle size, respectively. UV–Vis Differential Reflectance Spectroscopy (UV–Vis DRS; Analytikjena, Specord 210 plus, Germany) and Photoluminescence Spectroscopy (PL; Avantes-Avaspec-2048, Netherlands) determined Eg value and e^–^h^+^ recombination rate, respectively. Electrochemical Impedance Spectroscopy (EIS) was utilized to survey the electrical conductivity, and the potential of CB (E_CB_) (OrigaFlex, France). UV–vis spectrophotometer (RayLeigh UV-2601, Beijing Beifen-Ruili Co.; China) was employed to measure the removal efficiency of MB.

### The assessment of catalytic performances

#### The MB decolorization efficiency and catalytic degradation mechanism

**Figure 1:**
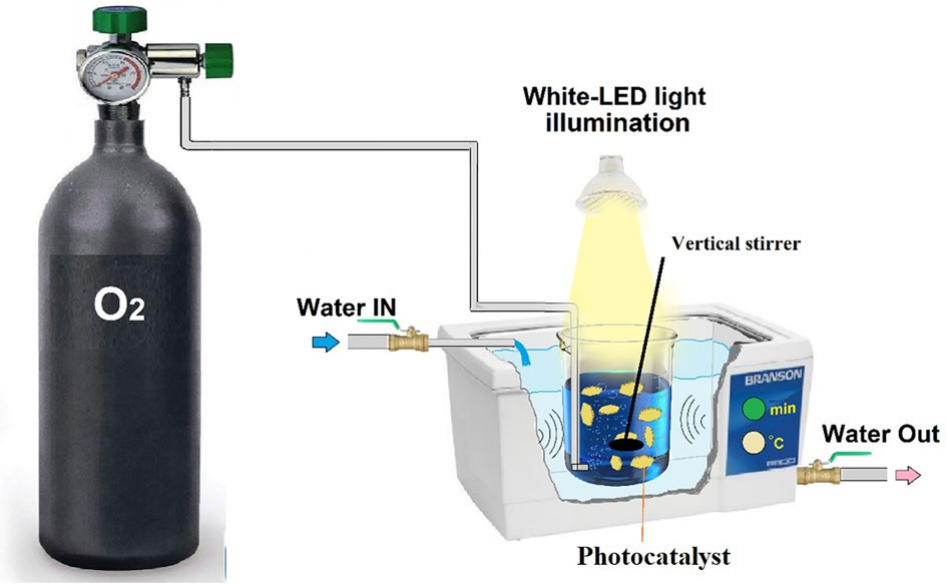
The schematic of the used sonophotocatalytic reactor.

The experiments were carried out at the specified dosage of catalyst (0.25, 0.5, 0.75, and 1 g L^−1^), the adjusted pH value (3, 5, 7, 9, and 11), and the desired initial MB concentration (10, 15, 20, and 25 mg L^−1^). Then, the suspension was agitated for 0.5 h in the non-illuminated (dark) condition; then, it turned irradiation sources on [white-LED lamp (30 W, 2800 Lumen) and or Ultrasonic bath (28 kHz, 100 W)] with or without opening Oxygen inlet and controlling temperature (Fig. 1). At specified time intervals (30, 45, 60, 75, 90, 105 and 120 min), 4 ml of the reactive suspension was removed. The suspension was then centrifuged at 4000 rpm. The decolorization efficiency of MB (DEM) was calculated using Eq. (1) where A0 and At are assigned to initial and final absorption of MB, respectively. According to Eq. (2), the degree of the MB mineralization [i.e., Total Organic Carbon (TOC) decay efficiency] was checked by a TOC analyzer (Shimadzu UV-160, German), where TOC_t=0_ and TOC_t=x_ is ascribed to the TOC value in the MB degradation process before running and after the specific time, respectively.

1 $$\mathrm{DEM }({\%})=\left[1-\frac{{\mathrm{A}}_{\mathrm{t}}}{{\mathrm{A}}_{0}}\right]\times 100$$

2 $$\mathrm{TOC\, decay\, efficiency }({\%})=\frac{{\mathrm{TOC}}_{\mathrm{t}=0}-{\mathrm{TOC}}_{\mathrm{t}=\mathrm{x}}}{{\mathrm{TOC}}_{\mathrm{t}=0}}\times 100$$

To detect the reactive species that generate in the catalytic reactions, 4 m.mol of three typical scavengers (i.e., BQ, BA, and AO preferred as the scavengers of ${\mathrm{O}}_{2}^{\cdot-}$, ${\mathrm{HO}}^{\cdot}$, and hole (h^+^), respectively) separately used in the sonocatalytic degradation of MB under the optimum condition. According to Eq. (1), the effectiveness of each active species on the MB degradation process was studied.

### Electrochemical measurements

All electrochemical measurements were accomplished in a three-electrode cell. FTO electrodes deposited with the samples as the working electrode, a platinum wire as the counter electrode, and Ag/AgCl [saturated KCl (3 M)] as a reference electrode respectively. All tests were conducted in a Na_2_SO_4_ (0.1 M) aqueous solution as the supporting electrolyte using electrochemical workstation (Origaflex/France OG 01A). For the preparation of the working electrodes, 200 mg of the obtained photocatalysts were mixed with a small amount of polyethylene glycol and distilled water. The mixture then blended in a mortar until the homogenous slurry obtained and spread onto the Fluorine-doped Tin Oxide (FTO) glass (1 × 1 cm^2^). The prepared films then were heated at 400° C for one hour in a muffle furnace.

The Electrochemical Impedance Spectroscopy (EIS) was carried out at a frequency range from 10 kHz to 100 mHz with a 10 mV AC voltage, which the Mott-Schottky plots obtained under AC potential frequency 1 kHz, and DC potential range from −1 to + 1 eV vs. Ag/AgCl under dark environment.

## Results and discussion

**Figure 2:**
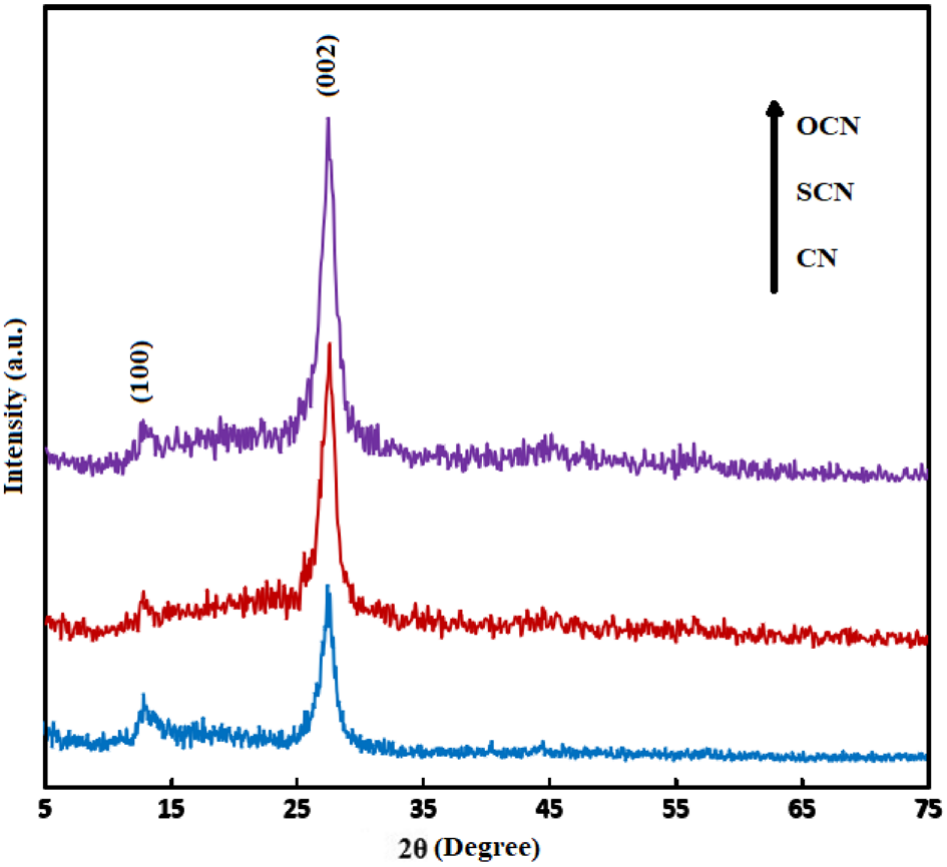
XRD patterns of the prepared samples.

As depicted in Fig. 2, the diffraction peaks located at around 13.1° (100) and 26.8° (002) attributed to the in-plane structural packing of tri-s-triazine units and the typical graphene-like stacking of the conjugated aromatic motif, respectively. Following JCPDS No. 87-1526, these plates are attributed to hexagonal graphitic materials, confirming the lamellar nature of g-C_3_N_4_^39^. The (100) peak positions of the modified samples had a negligible shift than CN, indicating that the Oxygen or Sulfur atoms were successfully replaced with Nitrogen and Carbon atoms in the tri-s-triazine units of g-C_3_N_4_. Besides this, the intensity of (100) peak weakened than CN, indicating that the in-plane repeating motifs enlarged. Meanwhile, the position of the (002) peaks was constant, illustrating the retention of the crystalline motif of g-C_3_N_4_. More importantly, the (002) diffraction peaks of the modified samples become sharper and narrower in comparison with CN, implying that the increased degree of polymerization of the precursors led to the higher crystalline structure of g-C_3_N_4_ in SCN and OCN than CN^23–25^. Scherer equation ($D=\frac{K\lambda }{\beta Cos\theta }$, where D, K, λ, β, and θ represent the crystal size, the shape constant value of 0.9, the X-ray radiation wavelength of 1.5418 Å, the full line width at half-maximum height, and the half of the scanning angle range, respectively) uses to calculate the crystalline size of particles. Hence, the index of (002) in CN, SCN, and OCN samples were selected for measuring D, which determined 17.3, 27.7, and 35.9 nm, respectively^40^. Furthermore, no obvious signal of impurities was detected, which is related to the high purity of the prepared catalysts. In summary, the findings suggest that non-metallic doped g-C_3_N_4_ samples were successfully produced and their higher crystalline nature was effectively enhanced by introducing different dopant atoms into the g-C_3_N_4_ lattice structure^41^.

The obtained FT-IR spectra of the synthesized samples are demonstrated in Fig. S1. The presented characteristic peaks at around 887 and 1200–1700 cm^−1^ originated from the deformation mode of N–H and the stretching vibration of heptazine heterocyclic ring units, respectively^19, 41, 42^. In particular, two peaks located around 800–890 cm^−1^ refer to the out-of-plane breathing mode of tri-s-triazine units. The intensity of these peaks was more vigorous in SCN and OCN than in CN, indicating that the crystalline network of g-C_3_N_4_ had become widespread via the successful insertion of Oxygen or Sulfur atoms in the g-C_3_N_4_ framework, agreeing well with the results of the acquired XRD patterns^41, 43^. In addition, the broader peak at 3100–3500 cm^−1^ corresponds to –OH stretching states and uncondensed amine groups, while the decrease in the intensity of this peak is attributed to the greater degree of polymerization of the precursors in SCN or OCN than in CN^36^. Moreover, the stretching vibration of the C–O–C peak extremely overlapped at around 1240 cm^−1^^41^. More importantly, decreasing the intensity of the –NH_x_ band is due to a more crystalline nature in SCN and OCN than CN, revealing that the most active sites probably do not exist in the lateral edges of SCN or SCN, effectively can lead to diminishing the long-term adsorption of organic pollutant molecules to dominate the catalytic activity^21–23^.

**Figure 3:**
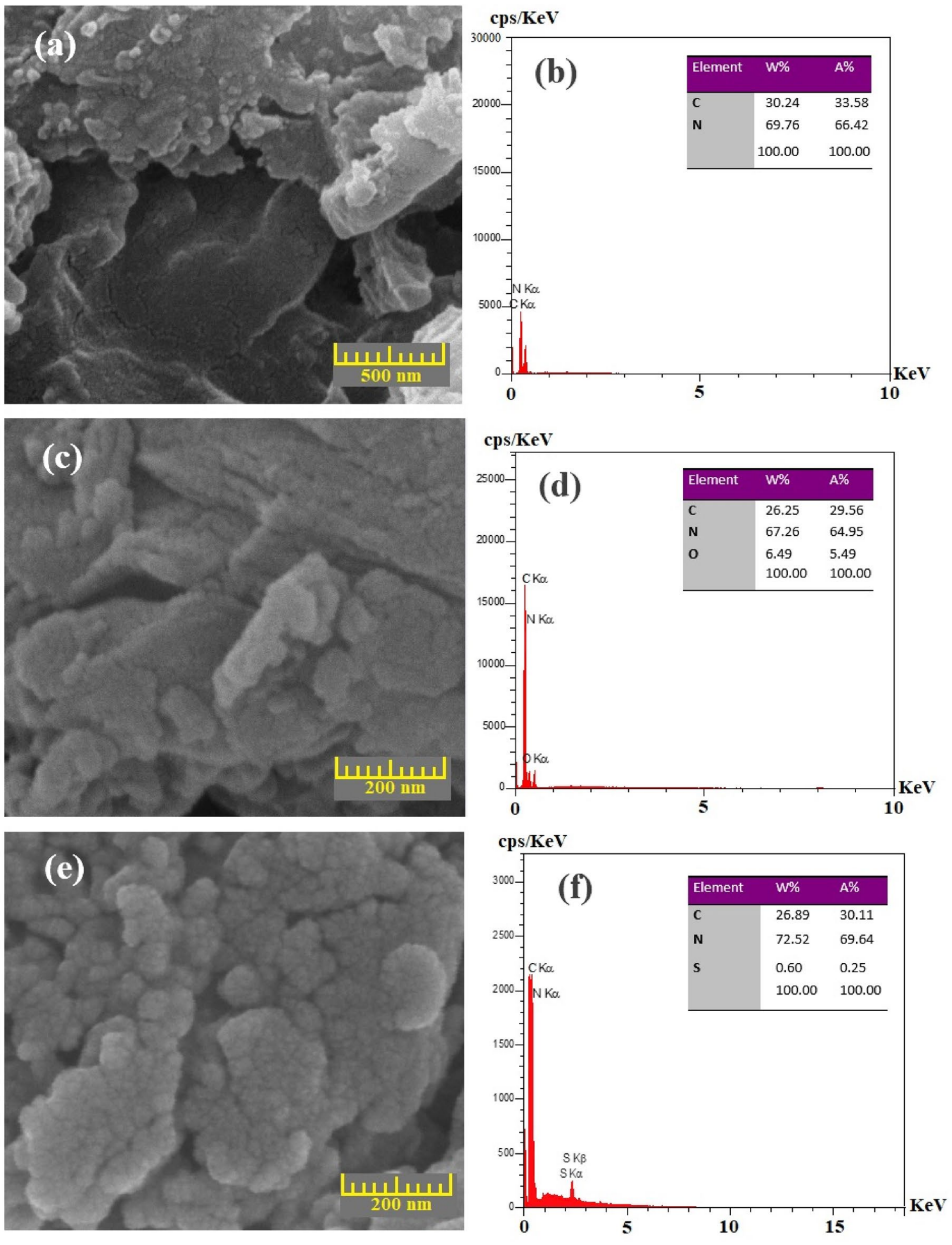
FE-SEM images of the synthesized samples ((**a**) CN, (**c**) OCN, and (**e**) SCN), and EDS spectra with the elemental composition of (**b**) CN, (**d**) OCN, and (**f**) SCN.

The microscopic structural and surface morphology of CN, OCN, and SCN were investigated by FE-SEM. The FE-SEM images in Fig. 3a, c, e show that the structure of the prepared samples includes large grains and two-dimensional bulky sheets with irregular blocks and non-uniform morphology. Unlike CN, which has nearly smooth sheets with sizes of about a few micrometers, SCN and OCN show semi-bulky fractions with smaller sheet size, which is probably the result of the polymerization process. In fact, the thermal conditions of the precursors for the preparation of SCN and OCN have led to changes in the size and shape of the sheets. Smaller particle size and higher porosity network of modified samples compared to CN can lead to an increase in the light-based performance of these samples. In addition, according to the information in Fig. 3a, c, e and Fig. S2, the particle sizes of SCN and OCN are in the range of one micrometer, which shows that a significant reduction in the length and thickness of the modified samples has occurred compared to CN. In fact, when melamine and oxalic acid or thiourea were used as precursors, volatile H_2_O or H_2_S gases created inorganic nanocrystals with a microporous structure in the g-C_3_N_4_ framework. In addition, the release of these gases can exfoliate carbon nitride sheets with more wrinkles and irregular shapes. Therefore, these smaller sizes of carbon nitride can lead to increased mass transfer phenomenon in SCN or OCN compared to CN. And the catalytic responses of photocatalysts can be improved^19, 30, 44–49^.

Figure 3b, d, f revealed that Oxygen or Sulfur elements successfully doped in g-C_3_N_4_ structure as OCN or SCN, respectively. Besides this, the prepared samples had high purity without disturbing elements, helping the prepared samples can display the truth of catalytic activities without disruptive impurities^44^. Moreover, a comparison between SCN and OCN evinced that the Oxygen content was more than Sulfur, originating from the truth that AO exerted a potent force for inserting Oxygen atoms into the g-C_3_N_4_ structure. Then, it changed the elemental composition of the g-C_3_N_4_ motif during the polymerization step, interpreting the stronger electronegativity of Oxygen than Sulfur for doping into the g-C_3_N_4_ structure^19, 41^.

**Figure 4:**
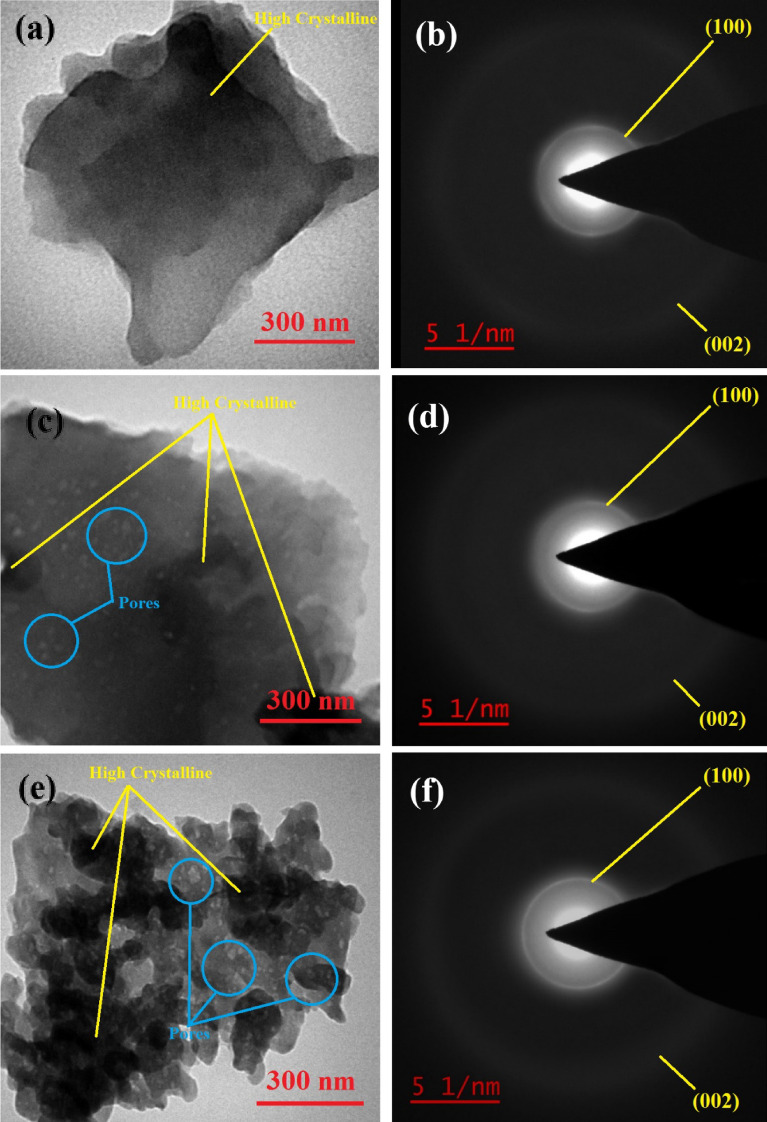
HR-TEM images of the synthesized samples ((**a**) CN, (**c**) OCN, and (**e**) SCN), and SAED pattern of the prepared samples ((**b**) CN, (**d**) OCN, and (**f**) SCN).

As shown in the HR-TEM images in Fig. 4a, c, e, the as-prepared samples showed a graphite-like shape with a layered configuration. In addition, the planar structure of CN was transformed into a porous structure in SCN or OCN due to the extraction of off-gases during the intense polymerization of melamine or AO^20^. It is worth noting that the porous structure improves the contact between the surfaces of the catalyst and reactant molecules and leads to the improvement of the catalytic activities of SCN or OCN compared to CN^43^. Significantly, the dark area in the images of SCN and OCN shows the higher crystallinity framework of g-C_3_N_4_, which indicates that more tris-*s*-triazine units are uniformly arranged in these samples than in CN^38, 50^. According to Fig. 4b, d, f, the absence of bright spots in the SAED patterns indicates that these samples have a crystalline nature. In addition, the identified rings match well with the double peaks obtained in the XRD patterns of Fig. 2.

Based on IUPAC classification methods, SBET and pore size distribution in the synthesized catalysts were determined by BET-BJH techniques. The adsorption–desorption isotherms of N_2_ gas are shown in (Fig. S3). Information is well presented on the type IV isotherm and H3 residual loops (P/P0 < 0.98), which are associated with slit-like pores resulting from the stacking state of the nanosheets^22, 51^. In addition, the obtained isotherms of the non-metal-doped samples shifted toward higher volume compared with CN, proposing that using AO in polycondensation of Melamine presumably led to producing more exhaust gases that concluded to enhancing S_BET_ and the mesopore nature. With a more detailed observation of the statistics summarized in Table 1, the SBET is gradually enlarged and the presence of mesoporous structures (pore size less than 50 nm) sufficiently increases the number of active sites and diffusion channels, resulting in active centers for Provides quick access to reactants^41, 43^. In addition, mesoporous structures increase the scattering and multiple reflection of received light and improve the light-dependent responses of catalysts^51^. According to the SBET obtained from SCN and OCN samples, it was concluded that the g-C3N4 non-metallic doping process shows that the catalytic activity is improved, which leads to high sonophotocatalytic performance. More significantly, the relatively low SBET and small pore volume increase the long-term stability of the prepared catalysts^52^.

**Table 1:** The attained data from adsorption–desorption isotherms.

Sample	S_BET_ (m^2^ g^−1^)	Total pore volume (cm^3^ g^−1^)	Mean pore diameter (nm)
CN	7.351	0.02922	15.89
SCN	8.814	0.08127	36.96
OCN	11.248	0.04546	16.47

**Figure 5:**
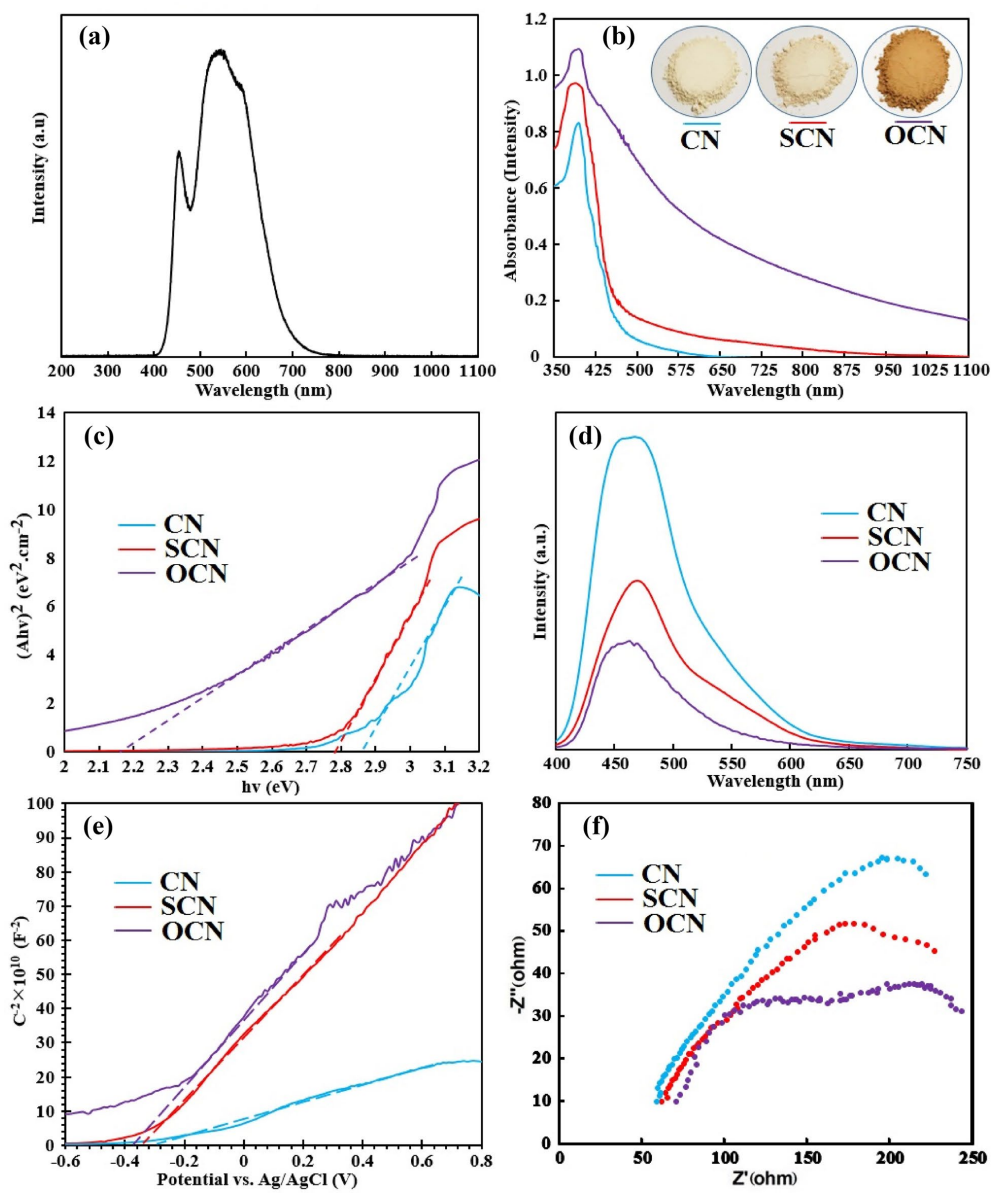
(**a**) The irradiated spectrum of the used white-LED lamp along with the visual color of the as-prepared samples, (**b**) UV–Vis DRS spectra, (**c**) the obtained Tauc plots from the DRS spectra, (**d**) PL spectra, (**e**) Mott–Schottky plots, and (**f**) Nyquist plots.

It is worth noting that the white-LED light source used in this experiment had the ability to emit in the visible light region (λ ≥ 420 nm) (Fig. 5a), the light collection spectrum of the prepared catalysts effectively matches this spectrum. Diffuse reflectance spectra of the as prepared samples are plotted in Fig. 5b. A strong increase in light absorption between 350 and 420 nm was detected for the prepared OCN and SCN samples compared to CN, which implied that increasing mesoporous nature could affect the photo-responses in the high-energy levels of the received light^53^. Besides this, the absorption edge of these modified samples presented a little blue-shift compared to CN, which may be ascribed to the modified van der Waals interactions between tri-*s*-triazine units due to progressing layer-by-layer exfoliation and decreasing particle size under harsh calcination^53^. Remarkably, the light absorption spectrum of OCN and SCN samples severely red-shifted at the entire visible-light spectrum, originating from the larger atomic radius of Sulfur or Oxygen atoms, and loosely bonding electrons of the dopant atoms in the π-π conjugated system. All of these benefits can lead to facilitating the n → π* transition in the sp^2^ hybridization, increasing the generation of the photo-excited of e^–^h^+^ pairs, and further charge distribution in the g-C_3_N_4_ domain^16, 38, 54^. In addition, the visual investigation reveals that the color change from pale yellow to dark brown in CN to OCN, respectively; signifying that the non-metal doped samples can absorb a broader range of the received light^38, 55^.

The band gaps obtained from the Tauc plot, using the Kubelka–Munk function are shown in Fig. 5c, the liner area extrapolated to determine Eg, which obtained 2.17, 2.79, and 2.86 eV for OCN, SCN, and CN, respectively. Therefore, the narrower Eg of OCN and SCN compared to CN, shows that OCN and SCN samples can absorb more solar energy to produce more electrons and photon-hole pairs, which leads to the improvement of the optical activity of the samples^52^.

PL spectra display the photo-generated luminescent intensity of the catalysts, introducing the charge carrier of separation, excitation, recombination, or even migration phenomenon. As known, the defect sites could act as the recombination center of the photo-induced charges. According to the PL quenching theory, if a photocatalyst demonstrates the low-intensity peak of PL, signifying it has a low recombination probability of photo-excited charges, and a high crystallinity nature. As depicted in Fig. 5d, under the wavelength illumination of λ_excitation_ = 355 nm and the expansive emission peak of the λ_emission_ ~ 470 nm, the CN had higher intensity than SCN or OCN. the high crystalline frameworks of the modified samples could have special features, such as the efficient separation ability, and significantly prevent the direct reuniting of charge carriers. Compared with CN, the low peak intensity of PL in SCN or OCN samples denoted that the non-metal atoms were successfully inserted in the g-C_3_N_4_ network, which could probably facilitate the charge transport across the photocatalyst interface. In conclusion, the superior suppressed charge carriers' recombination could donate the enhanced photocatalytic performance in the modified samples^18–23, 56^.

Mott-Schottky plots use to determine the flat band (FB) potential of photocatalysts, thereby meticulously calculating their CB and VB positions. According to Fig. 5e, the FB of CN, SCN, and OCN obtained −0.29, −0.34, and −0.38 V vs. the Ag/AgCl electrode, respectively. In addition, the positive slope approves the n-type conductivity of the prepared samples. As known, the CB potential of the n-type semiconductors has the vicinity to FB (exactly from −0.1 to −0.4 eV); and, the gap between the CB and the FB is usually selected −0.2 eV^41^. Therefore, the CB of CN, SCN, and OCN were estimated at around −0.49, −0.54, and −0.58 eV vs. the Normal Hydrogen Electrode (NHE), respectively. As the displayed Tauc plots in Fig. 5(c), the Eg of CN, SCN, and OCN were 2.86, 2.79, and 2.17 eV, respectively, resulting in the VB potential being calculated as + 2.37, + 2.25, and + 1.59 eV vs. NHE for the samples of CN, SCN, and OCN, respectively. The characteristics of the Eg revealed that SCN and OCN probably represent better photo-dependent performances than CN.

Figure 5f, shows the EIS analyzes to evaluate the resistance and charge transfer in CN, OCN, and SCN. The diameter of the arc radius in the Nyquist diagrams indicates the efficiency of charge transfer. As seen in Fig. 5f the smallest diameter of the arc radius belongs to the OCN, which shows that it has a low resistance to charge transfer and a high ability to separate and transfer e/h + pairs. Therefore, OCN and SCN are expected to have better photocatalytic performance compared to CN^30, 43, 44^.

**Figure 6:**
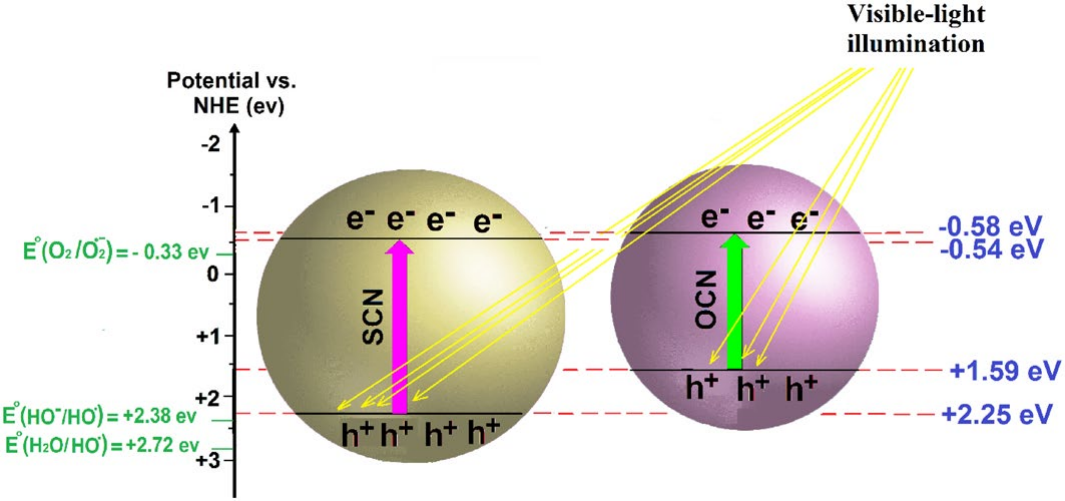
The position of CB and VB in SCN and OCN for performing visible-light-driven reactions in sonophotocatalysis.

Based on the aforementioned analysis, a plausible photocatalytic-based mechanism for the MB degradation using SCN or OCN was exhibited in Fig. 6. According to Fig. 5c, e, the E_CB_ of SCN and OCN were obtained at −0.54 and −0.58 eV, respectively; and, the E_VB_ of SCN and OCN were calculated + 2.25 and + 1.59 eV, respectively. Furthermore, E^o^ (H_2_O/${\mathrm{HO}}^{\cdot}$) and E^o^ (${\mathrm{HO}}^{-}$/${\mathrm{HO}}^{\cdot}$) are equal to + 2.72 eV and + 2.38 eV, respectively; which were more positive than the E_VB_ of the catalysts. It could cause generating h^+^ in the VB of catalysts to be unable to oxidize water molecules for producing ${\mathrm{HO}}^{\cdot}$. Moreover, the CB of catalysts was more negative than E^o^ (O_2_/${\mathrm{O}}_{2}^{\cdot-}$) = −0.33 eV, which could progress the transformation of O_2_ into ${\mathrm{O}}_{2}^{\cdot-}$ on the CB of catalysts^18, 52^.

### Assessing the effectiveness of the operational variables on MB decolorization

#### Screening the effect of catalyst's type and the utilized process

As known, the uncondensed-amine groups of g-C_3_N_4_ are strong active sites for pollutant absorption ability; therefore, it should be minimal for accelerating mass transportation over active centers' catalyst without long-term binding to the catalyst surface. As displayed in Fig. S4 and Fig. S1, CN had the most absorption of MB, arising from more amine groups that could create strong Hydrogen-bonding between MB molecules and g-C_3_N_4_ particles. On the contrary, the high-crystalline samples (i.e., SCN, and OCN) revealed better photocatalytic performances that could originate from the fewer uncondensed-amine groups, and the higher π–π electrostatic interactions between aromatic sections of MB and g-C_3_N_4_. Then, a hybrid process of US and photocatalysis exhibited the accelerated degradation of MB due to the US cavitation causing turbulence near the photocatalytic particles^57–60^. Therefore, this hybrid could have some benefits for photocatalysis, such as improving the photo-dependent capabilities of catalysts via the deaggregation of particles, continuously cleaning the catalyst's surface to avoid the accumulation of pollutants, accelerating the generation of ROS through the sonoluminescence phenomenon, and promoting mass transfer from the solution to the catalyst surface. Ultimately, the DEM followed the sequence: absorption < sonocatalysis < photocatalysis < sonophotocatalysis; and, the samples of OCN and SCN showed the best sonophotocatalytic activities compared with CN^9, 56–58^.

#### The initial concentration of MB

**Figure 7:**
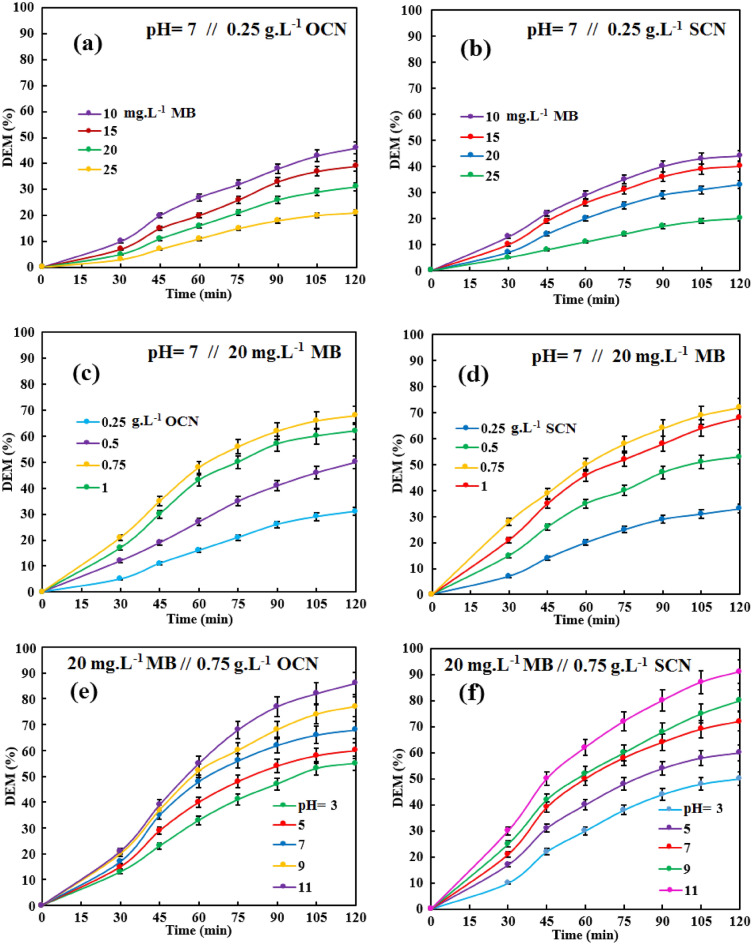
The effect of the operational variables: (**a,b**) the initial concentration of MB, (**c,d**) the catalyst dosage, and (**e,f**) the initial pH. In all experiments, the flow of O_2_ was interrupted; and, the temperature was held at about 21 °C.

As depicted in Fig. 7a, b, it can be found that the sonophotocatalytic MB degradation follows the order: 10 > 15 > 20 > 25 mg L^−1^ of MB, revealing that the higher concentration of MB can extremely impede the sonophotocatalytic performances. The DEM experienced a decreasing trend by increasing the initial concentration of MB, which could be referred to (I) As the Beer-Lambert law, increasing the turbidity of the solution prevents light penetration into the dye solution. (II) The generated ROS is approximately similar when the primary dosage of the catalyst is constant; whereas, the efficiency of the catalytic-oriented processes for producing new ROS diminished by occupying active sites in the higher concentration of MB and its by-products. Therefore, the argumentative MB concentration is considered as 20 mg L^−1^^5, 9^.

#### The catalyst dosage

As it is known, the efficiency of photocatalysis and sonocatalysis is strongly dependent on the catalyst dosage. Increasing the catalyst dosage can increase active sites, improve light utilization, and enhance ROS production. As shown in Fig. 7c,d, DEM increased drastically with increasing SCN or OCN dosage from 0.25 to 0.75 g L^−1^. A higher catalyst dose (i.e. 1 g L^−1^) was not able to enhance the sonophotocatalytic performance, indicating that the complementary role of US leads to the acceleration of the photocatalytic process. From a photocatalytic point of view, the turbidity reached its maximum at 0.75 g L^−1^ SCN or OCN, the excessive amount of catalyst may shield the light, thus decreasing the photon absorption of the photocatalysts. Therefore, US did not have much ability to maintain the upward trend of DEM, and it seems that US can only help to stimulate the catalyst charge during sonophotocatalysis^9^.

#### The initial pH

The vital variable that could determine the rate of catalytic-based reactions is the initial pH, which is affecting on the surface charge of the catalyst, the aggregation rate of particles, the rate of mass transfer rate into the diffusion layer across the catalyst surfaces, the type of catalytic reactions, and the electrochemical interactions between reactants and catalyst. As known, all as-prepared samples have the many –NH_x_ groups and the wide-spread aromatic network that could form the Hydrogen-bonds and π–π interactions between the MB molecules and the catalyst surface, respectively^9, 56^. Furthermore, the obtained ζ potential of CN, SCN, and OCN were −20.5, −12.6, and −6.71 mV, respectively, which correlates with the electronegativity of Oxygen, Nitrogen, Sulfur, and Carbon elements are 3.44, 3.04, 2.58, and 2.55, respectively; implying that the dopant with the higher electronegativity could easily insert into the g-C_3_N_4_ network, and increase the positive charge density^61–63^. Hence, the low negative charge of OCN and SCN could cause low pH sensitivity and weak electrostatic interactions between the cationic molecules of MB and the catalysts; thereby, the high basic pH could effectively ionize functional groups and increase the amount of negative surface charge. Thus, when the initial pH was adjusted to 11, the DEM improved due to the well-absorbed MB molecules that can effectually react on the highly negative-charged surface of catalysts (Fig. 7e, f)^9^.

### The kinetic investigation of the operational variables in the MB decolorization

The kinetic mechanism of the pollution degradation has complexities that can be assessed by calculating the pseudo-first-order (−Ln (A_t_/A_0_) = kt) and the pseudo-second-order ((1/A_t_) − (1/A_0_) = kt) equations, which “k”, “t”, “A_0_”, and “A_t_” are the apparent rate constant, the reaction time, the initial absorption of the MB-polluted solution, and the instantaneous absorption of the MB solution, respectively. The regression coefficient (R^2^) and k could calculate by evaluating the time-based plot of “−Ln (A_t_/A_0_)” or “(1/A_t_) − (1/A_0_)” towards the pseudo-first-order or pseudo-second-order reaction, respectively. Based on the obtained results in Fig. 7, the kinetic studies were accomplished. In the presence of the OCN sample (Table S1, and Fig. S5), the acquired results displayed that all variables had the highest number of R^2^ > 0.98 in pseudo-first-order. Meanwhile, for the SCN sample (Table S2, and Fig. S6), the parameter of the initial MB concentration only had the highest R^2^ > 0.98 in the pseudo-second-order, and the other variables followed the pseudo-first-order mechanism. Overall, all drawn of “−Ln (A_t_/A_0_)” had a nearly linear trend from beginning to end rather than “(1/A_t_) − (1/A_0_)”, confirming that the sonophotocatalytic reactions of the MB degradation followed the pseudo-first-order kinetic mechanism at presence OCN or SCN. Apart from the desired amount of MB concentration (i.e., 20 mg L^−1^), the other ideal variables (i.e., the catalyst dosage = 0.75 g L^−1^ and pH 11) had a remarkable agreement with the largest k, offering that the maximum mass transportation could successfully occur by sonophotocatalysis at the optimized values of the operational conditions^8, 64^.

### The sonophotochemical mechanism and the stability of SCN and OCN to degrade MB

**Figure 8:**
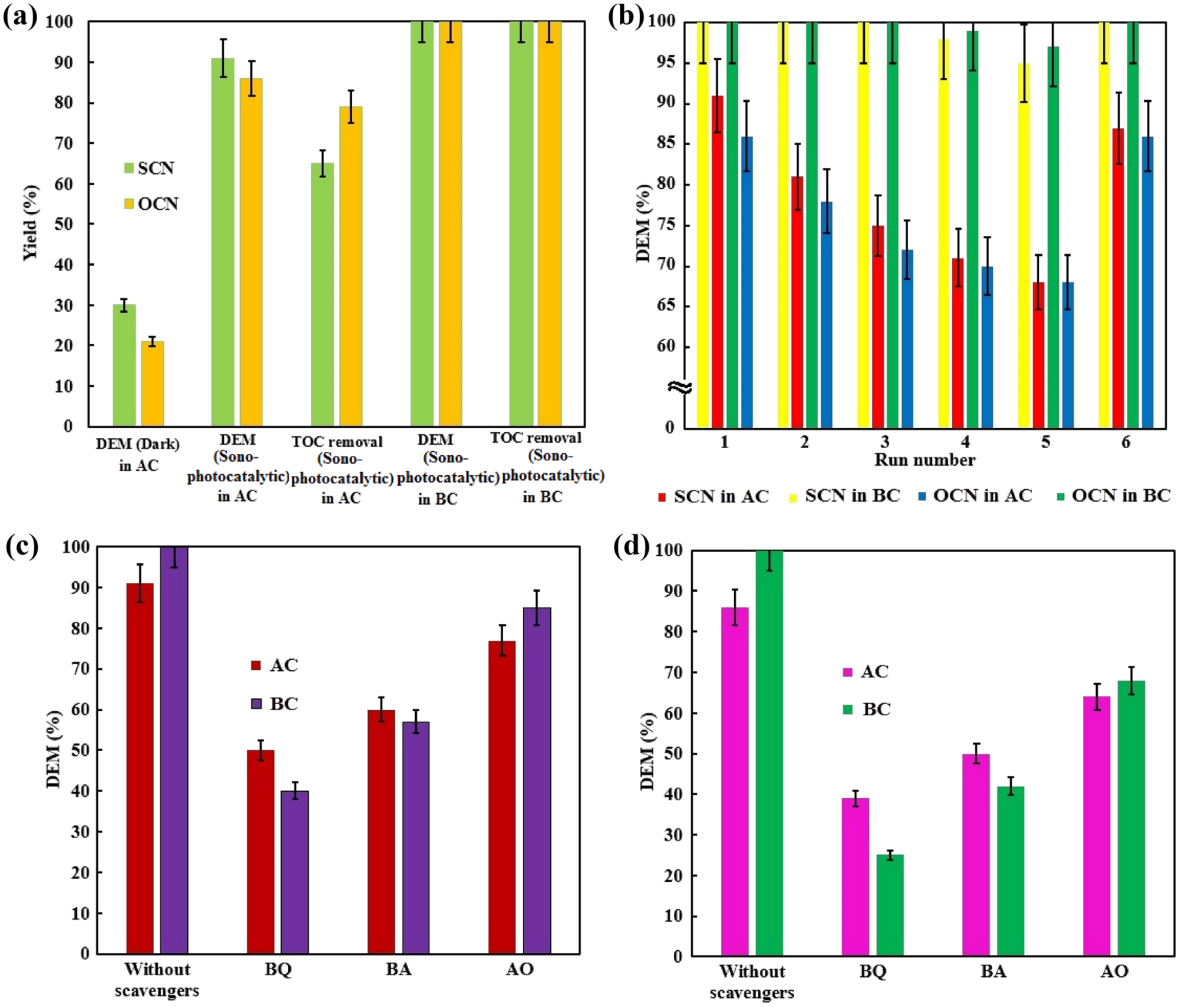
(**a**) Screening the portion of the processes along with showing the effect of injecting O_2_ gas along with spontaneous raising temperature in sonophotocatalytic degradation of MB, (**b**) the reusability tests for SCN and OCN, (**c**) the scavenging experiments over SCN, and (**d**) the scavenging experiments over OCN. (AC: without enhancers (without O_2_ injection + holding temperature about 21 °C); and, BC: with enhancers (injecting 0.25 mL s^−1^ O_2_ + spontaneous raising temperature without control)).

The DEM results obtained for SCN and OCN show that the combination of photocatalyst and sonocatalyst improves the catalytic process, (Fig. S4 and Fig. 7). Absorbed energies (Eqs. (3)–(14))^4, 8, 9, 64–66^. To ensure sufficient mineralization of the organic content, the use of boosters i.e., sufficient oxygen injection and spontaneous temperature increase during sonication can synergistically accompany each other to improve DEM and TOC decomposition (Fig. 8a). In the optimized sonophotocatalytic process, the observations can be justified by the following factors. (I) According to the Arrhenius theory, increasing the temperature during catalyst-based reactions can improve the reaction rate (Eqs. (3), (4) and (13)). (II) Apart from the catalyst surfaces that sites provide an activity to carry out the cavitation phenomenon, there are sites for the sonophotocatalytic process. Therefore, sufficient O_2_ dissolution can absorb the excited electrons and ${\mathrm{H}}^{\cdot}$ and affect the catalytic formation of ${\mathrm{O}}_{2}^{\cdot-}$ (Eqs. (5 and (6))^67^. (III) As hot spot theory, the irradiated energies could induce to excite the semiconductors for further producing e^–^h^+^ pairs (Eq. (4))^17^. (IV) As known, the lifetime and stability of ROSs generally is H_2_O_2_ > ${\mathrm{O}}_{2}^{\cdot-}$  > ${\mathrm{OH}}^{\cdot}$. Therefore, the additional consumption of O_2_ by photocatalysts could produce more ${\mathrm{O}}_{2}^{\cdot-}$ and H_2_O_2_, which consequently results in the photo-generated e^–^h^+^ pairs having enough opportunity for attacking H_2_O_2_ for the additional production of ${\mathrm{O}}_{2}^{\cdot-}$ and ${\mathrm{OH}}^{\cdot}$ (Eqs. (5)–(12))^67, 68^. Thus, these synergistic parameters can accelerate the excitation of charges from VB and prevent the accumulation of excited electrons in CB by promoting the activation of O_2_.

3 $${\text{H}}_{2} {\text{O}}\xrightarrow{{{\text{Ultrasond}}({\text{Cavitation}}{\kern 1pt} {\text{effect}})}}{\text{Light}}\left( {{\text{Sonoluminesence}}} \right) + {\text{Heat}}\left( {{\text{Hot}}{\mkern 1mu} {\text{spot}}} \right) + {\text{H}}^{ \cdot } + {\text{OH}}^{ \cdot }$$

4 $${\text{SCN}}\;{\mkern 1mu} {\text{or}}\;{\mkern 1mu} {\text{OCN}}\xrightarrow{{{\text{Eg}} < {\text{h}}\nu ({\text{White - LED}}{\mkern 1mu} {\text{light}} + {\text{Long}}{\mkern 1mu} {\text{wavelength}}{\mkern 1mu} {\text{irradiation}} + {\text{Heat}})}}{\text{e}}^{ - } + {\text{h}}^{ + }$$

5 $${\mathrm{O}}_{2}+{\mathrm{H}}^{\cdot}\to {\mathrm{O}}_{2}^{\cdot-}+{\mathrm{H}}^{+}$$

6 $${\mathrm{O}}_{2}+{\mathrm{e}}^{-}\to {\mathrm{O}}_{2}^{\cdot-}$$

7 $${\mathrm{O}}_{2}^{\cdot-}+{\mathrm{H}}^{+}\to {\mathrm{HOO}}^{\cdot}$$

8 $${\mathrm{HOO}}^{\cdot}+{\mathrm{OH}}^{-}\to {\mathrm{O}}_{2}^{\cdot-}+{\mathrm{H}}_{2}\mathrm{O}$$

9 $${\mathrm{HOO}}^{\cdot}+{\mathrm{H}}_{2}\mathrm{O}\to {\mathrm{H}}_{2}{\mathrm{O}}_{2}+{OH}^{\cdot}$$

10 $$2 {\mathrm{HOO}}^{\cdot}\to {\mathrm{H}}_{2}{\mathrm{O}}_{2}+{\mathrm{O}}_{2}$$

11 $${\mathrm{H}}_{2}{\mathrm{O}}_{2}+{\mathrm{e}}^{-}\to {\mathrm{OH}}^{-}+{\mathrm{OH}}^{\cdot}$$

12 $${\mathrm{H}}_{2}{\mathrm{O}}_{2}+{\mathrm{h}}^{+}\to {\mathrm{O}}_{2}^{\cdot-}+2{\mathrm{H}}^{+}$$

13 $${\mathrm{H}}_{2}{\mathrm{O}}_{2}\to 2{\mathrm{OH}}^{\cdot}$$

14 $$\mathrm{MB}+({\mathrm{OH}}^{\cdot}/{\mathrm{O}}_{2}^{\cdot-}/{\mathrm{h}}^{+})\to \mathrm{ Decomposed\, products}$$

The reusability of catalysts is one of the essential criteria for the decontamination process of an aqueous environment, when long-term industrial application is considered. To investigate the superiority of SCN and OCN over each other, we evaluated their recycling performance and reusability in the sonophotocatalytic degradation of MB over six consecutive trials. After each cycle, the catalysts were collected, washed and dried at 90 °C for 5 h^69^. Separation of these catalysts was performed by centrifugation due to their particle size of about one micrometer, which is in good agreement with the results of Fig. 3 and Fig. S2^70^. In addition to sufficient recyclability, the efficiency of sonophotocatalytic degradation of MB was compared in them and the findings are shown in Fig. 8b. When the temperature was kept at about 21 °C and no oxygen was injected into the suspension in each run, the obtained DEM showed a significant decrease of about 22%. Meanwhile, the injection of 0.25 mL s^−1^ of oxygen gas together with a spontaneous increase in temperature (from about 21 to 33 °C due to US irradiation) caused the DEM efficiency to be maintained for several consecutive periods without losing the profound efficiency. This observation confirmed that oxygen injection can prevent catalyst aggregation and effectively drive Eqs. (5) and (6). The enhancement temperature could accelerate the heat-dependent reaction (Eqs. (3), (4) and (12))^71–73^.

From the first to the fifth run, a slight decrease in the photocatalytic performance of SCN and OCN was observed, which could be due to the adsorption of a small amount of MB molecules and their side products, including mineral ions or photosensitive hydroxides, on the catalyst surfaces. When these disturbing agents accumulate on the surface of the photocatalyst, they block the active sites of the catalyst, and as a result, less excited electrons and less amounts of ${\mathrm{O}}_{2}^{\cdot-}$ and ${\mathrm{HO}}^{\cdot}$ are produced. Sometimes these accumulated byproducts may scavenge ${\mathrm{HO}}^{\cdot}$ or form bonds that reduce the catalytic activities of SCN and OCN. Therefore, the catalysts were calcined at 400 °C for 45 min to remove the unwanted side products from the active sites, which can lead to the restoration of the catalyst’s performance. In fact, the heat treatment process is suitable for the decomposition of organic compounds, which can greatly clean the catalyst surfaces to reactivate the catalysts. Fortunately, in the sixth run, the sonophotocatalytic abilities were restored to close to the initial run. More importantly, the diffraction peaks in Fig. S7 show that both SCN and OCN maintain their intensity and state without the appearance of new peaks, and in fact, the high crystallinity of SCN and OCN can prevent the loss of sonophotocatalytic efficiency. Thus, it is worth mentioning here that the repeatability and recyclability of these catalysts can attributable to their excellent physicochemical features and optimizing conditions for promoting sonophotocatalytic reactions^74–76^.

As known, the photocatalyst can continuously produce reactive species over the surface of itself. Besides this, it believes that radicals discontinuously formed by the transient implosion of cavitation bubbles near the photocatalysts could reinforce the degradation efficiency of MB and its derivative molecules. As a result, the MB can decompose by h^+^, ${\mathrm{O}}_{2}^{\cdot-}$, and ${\mathrm{HO}}^{\cdot}$ in an ultrasound-accelerated photocatalysis; thereby, the scavenging trials were carried out to identify the dominant reactive species in the presence of AO, BQ, and BA, respectively^9, 73^. As depicted in Fig. 8c, d, the addition of BQ effectually led to inhibiting the MB decolorization, indicating that ${\mathrm{O}}_{2}^{\cdot-}$ played the determining role in the optimized sonophotocatalytic process. On the contrary, the DME did not significantly reduce after introducing AO, representing that h^+^ was not the primary active substance. In addition, at presence enhancers in the optimized condition, the effect of ${\mathrm{O}}_{2}^{\cdot-}$ and ${\mathrm{OH}}^{\cdot}$ was much higher than that of the h^+^. In addition, the majority of ${\mathrm{OH}}^{\cdot}$ generation can result from the indirect conversion of ${\mathrm{O}}_{2}^{\cdot-}$ and the cavitation effect (Eqs. (3) and (7–12)). In summary, the obtained results show the prominent role of ${\mathrm{O}}_{2}^{\cdot-}$ and then ${\mathrm{OH}}^{\cdot}$ in the removal of MB^77^.

## Conclusion

In summary, some simple modification methods for the fabrication of SCN and OCN were carried out through a one-pot non-metallic doping process under special polymerization conditions, which ultimately led to the enhancement of the optical and photocatalytic properties of SCN and OCN, such as reducing Eg and improving light utilization ability, and reduced recombination of charge carriers. So far, the applied synthesis modifications resulted in a safe, inexpensive and simple approach to fabricate highly crystalline nonmetallic g-C_3_N_4_ with good structural and surface properties. Then, DRS analysis showed that white-LED light has good harmonics for carrier excitation. Cold-white-low-power LED light showed suitable photocatalytic responses for MB reduction. According to Figure S8, with the synergy between US and photocatalyst, the use of US successfully promoted the photodegradation of MB under optimized conditions. Apart from the role of US in accelerating the excitation of charge carriers by the emission of long-wavelength radiation, the spontaneous increase in temperature (as the first enhancer) showed that the crossing of the activation energy barrier could be improved to facilitate the catalytic reactions. The second booster (i.e., O_2_ gas injection) synergistically with sonophotocatalysis, ultimately leads to an increase in MB mineralization efficiency by trapping the most excited electrons to produce ${\mathrm{OH}}^{\cdot}$ Therefore, the introduction of these enhancers into the sonophotocatalytic systems finally revealed the maximum capabilities of the designed heterogeneous AOPs. In conclusion, these practical implementations optimistically extended our insight into the preparation of long-lived g-C_3_N_4_-based catalysts and enhanced sonophotocatalytic performance for rapid decontamination of organic dyes under low-power radiation sources.

### Supplementary Information

Supplementary Information.

## Data Availability

All data generated or analyzed during this study are included in this published article (and its supplementary information files).
