# Supplementary material for: Preparation of high-crystalline and non-metal modified g-C3N4 for improving ultrasound-accelerated white-LED-light-driven photocatalytic performances
Source: Sci Rep. 2023 Sep 12;13:15079. doi: 10.1038/s41598-023-41473-y (PMC10497575; doi:10.1038/s41598-023-41473-y)
Supplement: Supplementary file 1 — Supplementary Information. [file 41598_2023_41473_MOESM1_ESM.docx]

**Supplementary data**

**Preparation of high-crystalline and non-metal modified g-C_3_N_4_ for improving ultrasound-accelerated White-LED-light-driven photocatalytic performances**

Abdolreza Tarighati Sareshkeh^1^, Mir Saeed Seyed Dorraji^*, 1^, Zhaleh Karami^1^, Saeedeh Shahmoradi^1^, Elnaz Fekri^1^, Hoda Daneshvar^1^, Mohammad Hossein Rasoulifard^1^, Denis N. Karimov^**, 2^

1-­ Applied Chemistry Research Laboratory, Department of Chemistry, Faculty of Science, University of Zanjan, Zanjan, Iran.

2- Federal Scientific Research Center «Crystallography and Photonics», Russian Academy of Sciences, Leninsky Prospekt 59, 119333 Moscow, Russia

*, **: Corresponding Authors' E-mail:

dorraji@znu.ac.ir (M.S. Seyed Dorraji)

dnkarimov@gmail.com (D. N. Karimov)

Additional Supporting Data

Table captions

**Table S1.** The calculated of k and R^2^ for operational parameters at presence OCN.

**Table S2.** The calculated of k and R^2^ for operational parameters at presence SCN.

Figure captions

**Fig. S1.** The FT-IR spectra of the prepared samples.

**Fig. S2**. DLS curves of as-prepared samples ((a) SCN, and (b) OCN) obtained based on particle size.

**Fig. S3.** The adsorption-desorption isotherms of the prepared samples.

**Fig. S4.** The effect of catalyst's type and the utilized process under primary condition (The initial pH= 7, Catalyst dosage= 0.25 g.L^-1^, and The initial concentration of MB= 10 mg.L^-1^).

**Fig. S5.** (a, c, and e) the pseudo-first-order and (b, d, and f) the pseudo-second-order reaction kinetic plot of MB degradation process at presence OCN.

**Fig. S6.** (a, c, and e) the pseudo-first-order and (b, d, and f) the pseudo-second-order reaction kinetic plot of MB degradation process at presence SCN.

**Fig. S7.** The XRD peaks for the fresh OCN and SCN (according to Fig. 2) along with the XRD peaks of them when 5 times used and reactivated for using in run 6^th^.

**Fig. S8**. The schematic of the summarized occurs on the sonophotocatalytic mineralization of MB.

**Table S1.** The calculated of k and R^2^ for operational parameters at presence OCN.

| Parameters | Levels | Pseudo-first-order | | Pseudo-second-order | |
| --- | --- | --- | --- | --- | --- |
|  |  | k (min^-1^) | R^2^ | k (L. mol ^-1^ min^-1^) | R^2^ |
| The MB concentration | 10 | 0.0054 | 0.9929 | 0.0067 | 0.9854 |
|  | 15 | 0.0045 | 0.9842 | 0.0034 | 0.9742 |
|  | 20 | 0.0034 | 0.9838 | 0.0020 | 0.9792 |
|  | 25 | 0.0022 | 0.9750 | 0.0011 | 0.9741 |
| The catalyst dosage | 0.25 | 0.0034 | 0.9838 | 0.0020 | 0.9792 |
|  | 0.5 | 0.0061 | 0.9926 | 0.0043 | 0.9760 |
|  | 0.75 | 0.0102 | 0.9831 | 0.0095 | 0.9791 |
|  | 1 | 0.0088 | 0.9782 | 0.0074 | 0.9772 |
| pH | 3 | 0.0072 | 0.9885 | 0.0054 | 0.9753 |
|  | 5 | 0.0083 | 0.9813 | 0.0067 | 0.9794 |
|  | 7 | 0.0104 | 0.9769 | 0.0096 | 0.9754 |
|  | 9 | 0.0132 | 0.9888 | 0.0145 | 0.9468 |
|  | 11 | 0.0176 | 0.9796 | 0.0255 | 0.8895 |

**Table S2.** The calculated of k and R^2^ for operational parameters at presence SCN.

| Parameters | Levels | Pseudo-first-order | | Pseudo-second-order | |
| --- | --- | --- | --- | --- | --- |
|  |  | k (min^-1^) | R^2^ | k (L. mol ^-1^ min^-1^) | R^2^ |
| The initial MB concentration | 10 | 0.0052 | 0.9797 | 0.0064 | 0.9855 |
|  | 15 | 0.0046 | 0.9788 | 0.0036 | 0.9834 |
|  | 20 | 0.0036 | 0.9805 | 0.0022 | 0.9833 |
|  | 25 | 0.002 | 0.9920 | 0.0010 | 0.9920 |
| The catalyst dosage | 0.25 | 0.0036 | 0.9805 | 0.0022 | 0.9833 |
|  | 0.5 | 0.0067 | 0.9884 | 0.0049 | 0.9876 |
|  | 0.75 | 0.0109 | 0.9958 | 0.0109 | 0.9798 |
|  | 1 | 0.0098 | 0.9957 | 0.0089 | 0.9768 |
| pH | 3 | 0.0063 | 0.9821 | 0.0045 | 0.9781 |
|  | 5 | 0.0082 | 0.9850 | 0.0067 | 0.9843 |
|  | 7 | 0.0112 | 0.9897 | 0.0111 | 0.9760 |
|  | 9 | 0.0136 | 0.9944 | 0.0161 | 0.9168 |
|  | 11 | 0.0205 | 0.9835 | 0.0388 | 0.8192 |


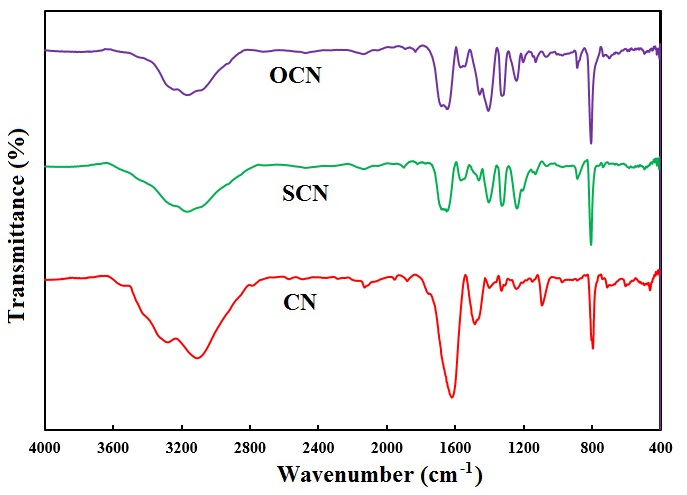


**Fig. S1.** The FT-IR spectra of the prepared samples.


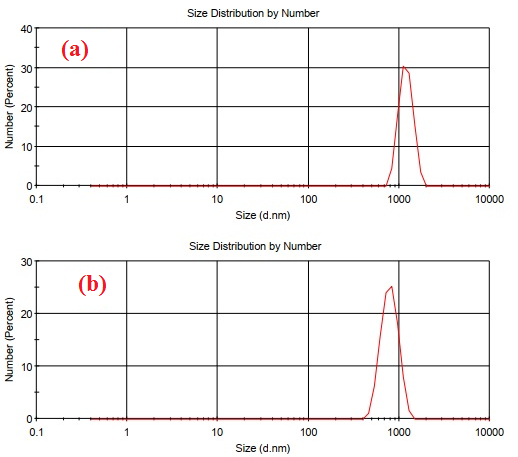


**Fig. S2**. DLS curves of as-prepared samples ((a) SCN, and (b) OCN) obtained based on particle size.


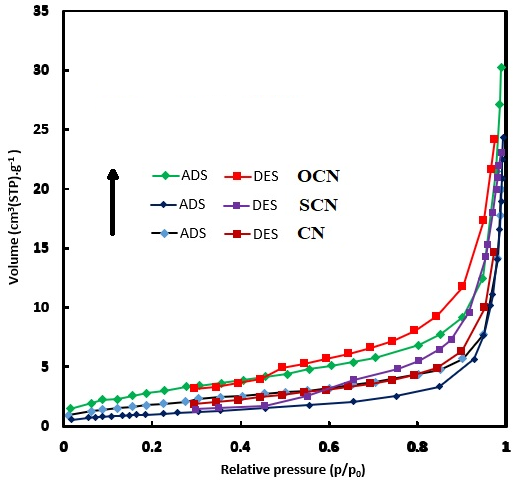


**Fig. S3.** The adsorption-desorption isotherms of the prepared samples.


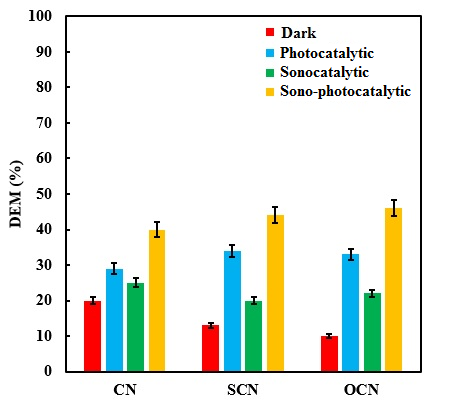


**Fig. S4.** The effect of catalyst's type and the utilized process under primary condition (The initial pH= 7, Catalyst dosage= 0.25 g.L^-1^, and The initial concentration of MB= 10 mg.L^-1^).


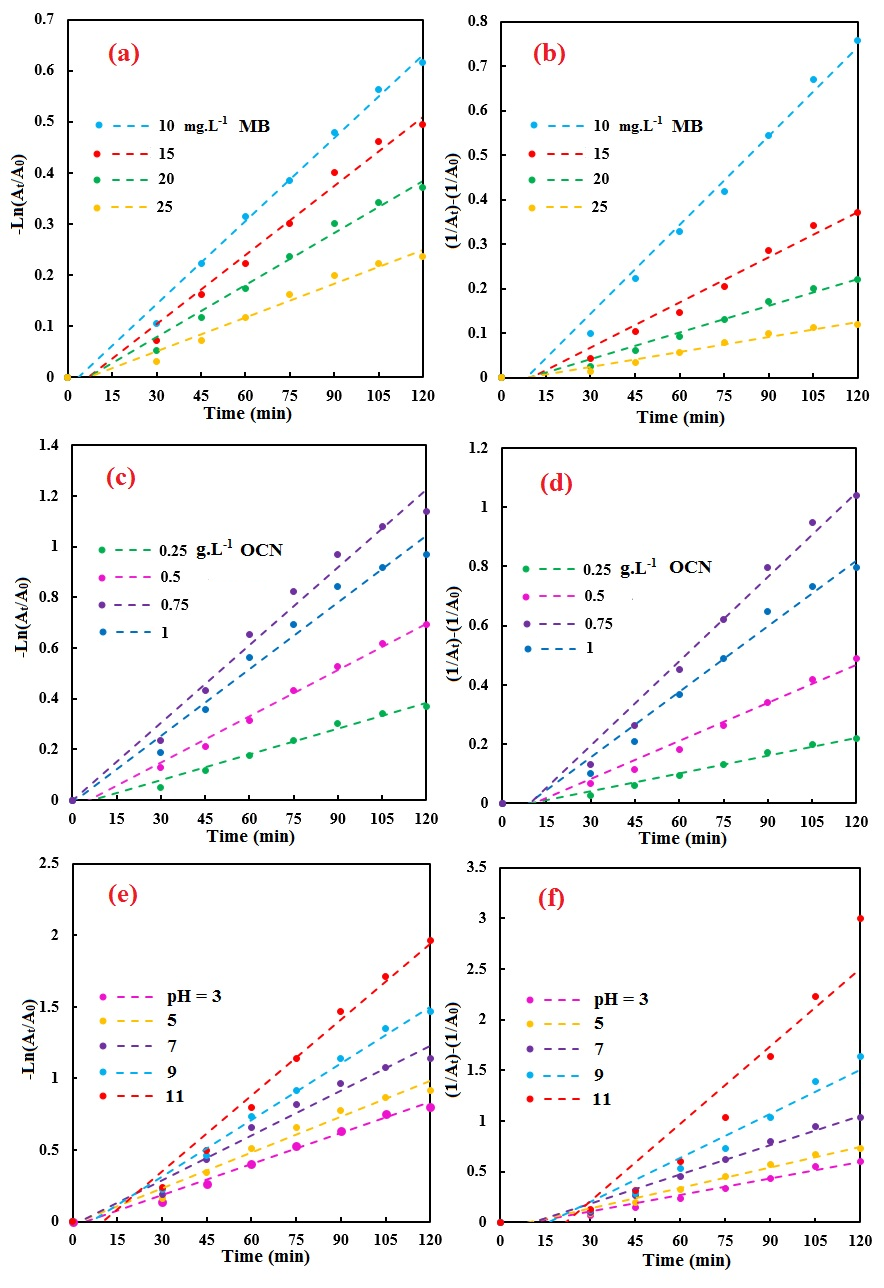


**Fig. S5.** (a, c, and e) the pseudo-first-order and (b, d, and f) the pseudo-second-order reaction kinetic plot of MB degradation process at presence OCN.


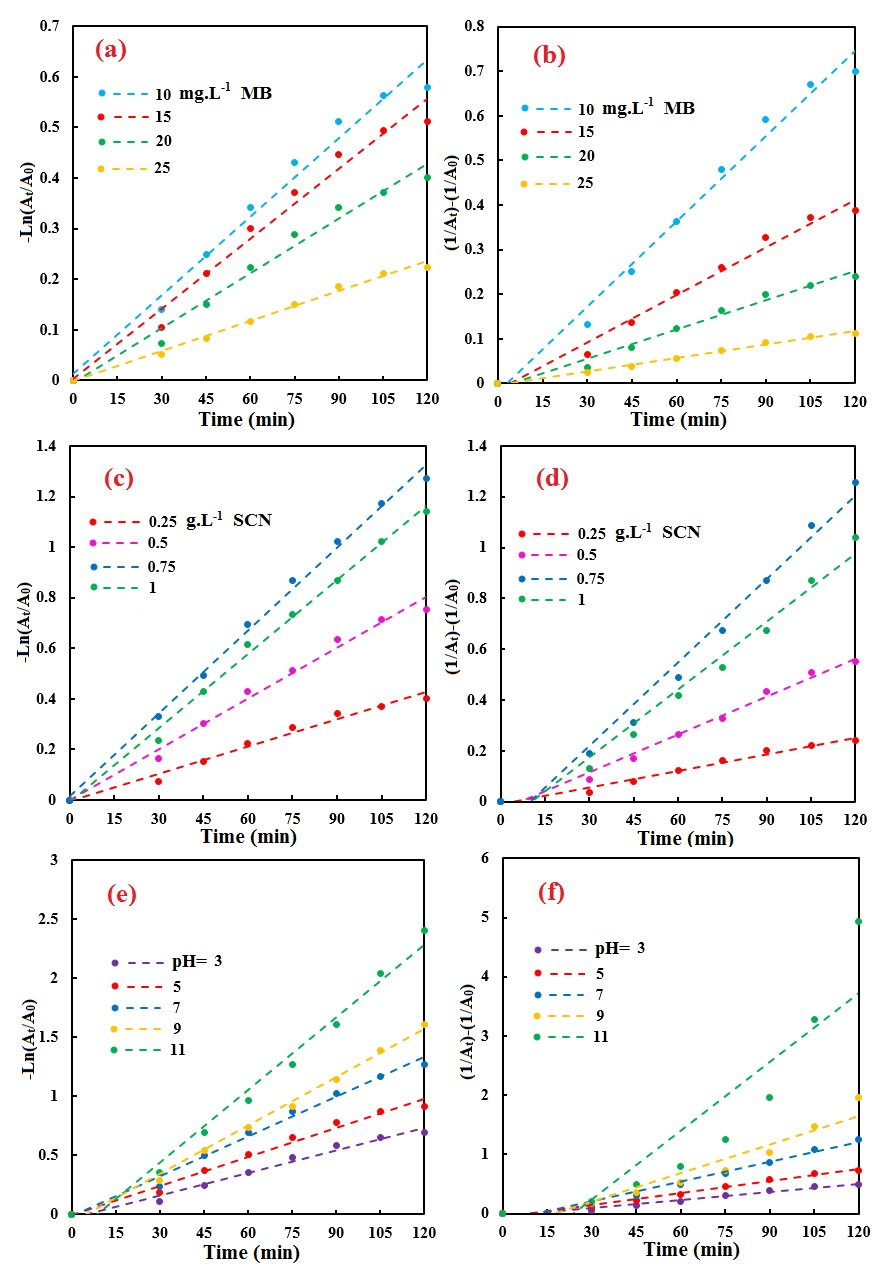


**Fig. S6.** (a, c, and e) the pseudo-first-order and (b, d, and f) the pseudo-second-order reaction kinetic plot of MB degradation process at presence SCN.

**
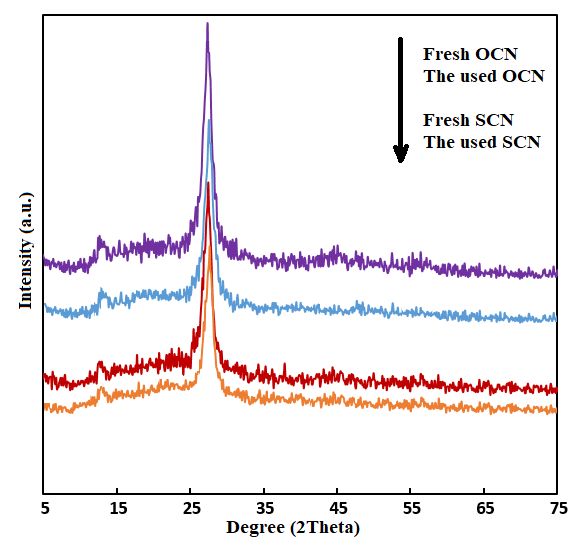
**

**Fig. S7.** The XRD peaks for the fresh OCN and SCN (according to Fig. 2) along with the XRD peaks of them when 5 times used and reactivated for using in run 6^th^.


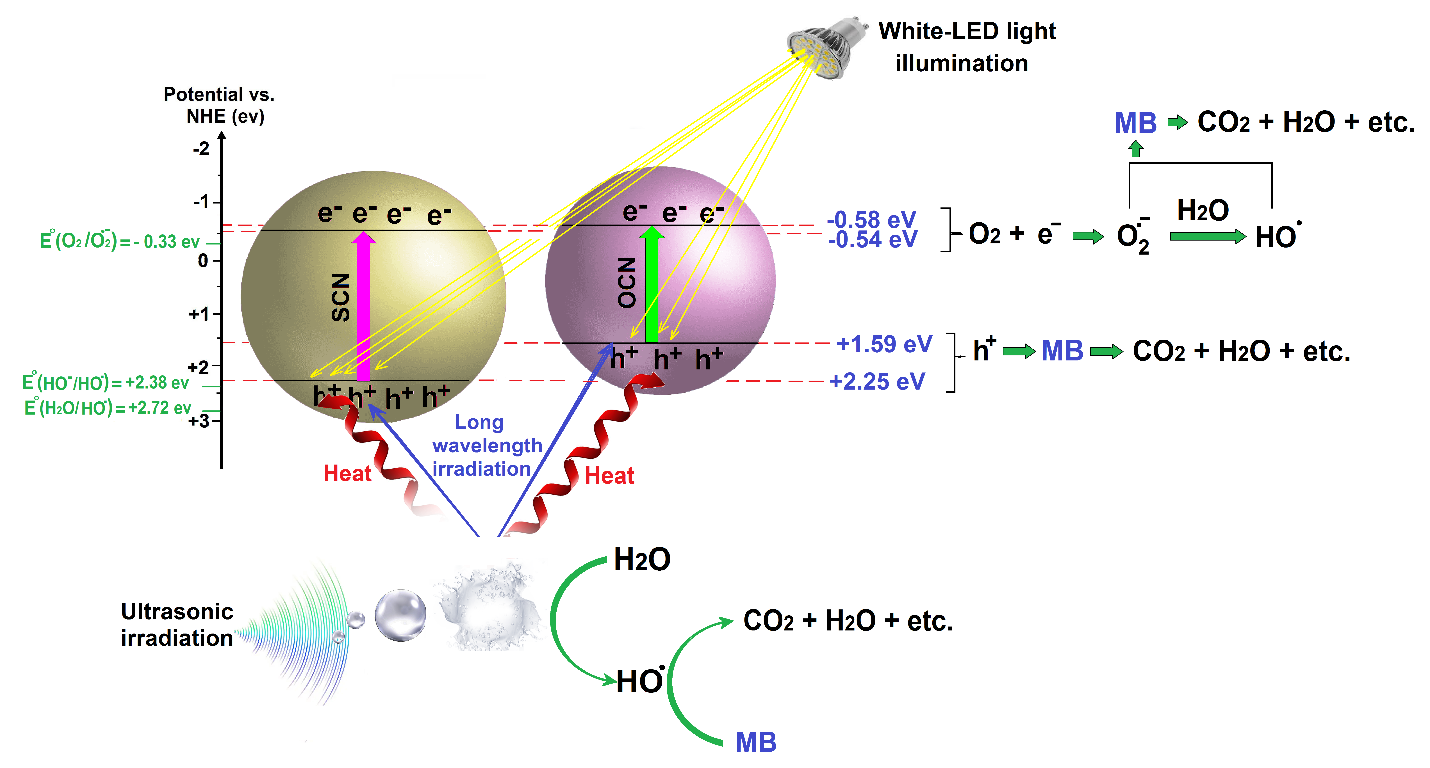


**Fig. S8**. The schematic of the summarized occurs on the sonophotocatalytic mineralization of MB.
